# Conformational Variations of a Key Enzyme Offer Clues to Cancer-Drug Resistance

**DOI:** 10.1371/journal.pbio.0040166

**Published:** 2006-05-02

**Authors:** Mary Hoff

The smooth operation of a cell requires that critical changes in molecular activity take place when and where they're supposed to. One family of enzymes, called tyrosine kinases, plays an important role in effecting these changes by attaching phosphate groups to other proteins in a process called phosphorylation. This chemical reaction initiates a tag team of signals that lead from a stimulus, through a series of intervening molecules, to a protein controlling a specific cellular process. To phosphorylate the recipient protein, a tyrosine kinase must first grab onto it with a highly specific lock-and-key connection—each kinase tailored to its unique target. This happens only when the kinase is in an active conformation—when the key has been twisted or bent by a signal from the previous molecule in the tag team into a conformation that will fit the shape of its target protein.

C-Abl is a tyrosine kinase that helps convey messages about cell growth and movement. Normally it does so only when it is activated by the preceding protein in the signaling pathway. But when cells possess a mutated form of c-Abl called BCR-Abl, the tyrosine kinase gets stuck in its active form, and sends the cell constant encouragement to grow. The miscommunication manifests itself as the blood cancer chronic myeloid leukemia, or CML.

A common treatment for CML is a drug called imatinib (known commercially as Gleevec or Glivec). Imatinib binds to a conformation of Abl (either c- or BCR-) in which a section called DFG has been rotated about 180 degrees from the active form, “DFG-Asp In,” to the inactive “DFG-Asp Out” conformation, preventing the tyrosine kinase from doing its phosphate-transfer job. But in some CML cells, imatinib doesn't work. A better understanding of the various conformations of the kinase domain of Abl will shed light on not only the molecule's function, but also how imatinib does—and sometimes doesn't—inhibit it.

Nicholas M. Levinson, Olga Kuchment, John Kuriyan, and colleagues explored the ins and outs of the kinase portion of normal and imatinib-resistant (mutant) Abl using crystallography, a process that makes it possible to take “snapshots” of proteins in various conformations. With the use of a novel synthetic bisubstrate analog inhibitor, the researchers found four previously undescribed forms of the kinase domain of Abl. They analyzed the position of various key amino acids and groups of amino acids within these conformations, as well as within a fifth, already-known conformation: that of Abl with imatinib attached.

In doing so, the researchers uncovered a big surprise: an inactive Abl conformation that differs dramatically from the rest of the conformations studied—and from all previously known conformations of Abl. This unprecedented inactive conformation was very similar to the inactive form of another kinase called Src, in which DFG is not flipped but another part of the kinase, an alpha helix, is swung out from the active conformation, known as αC-Glu In, into the inactive form, αC-Glu Out.

The researchers explored the functional significance of the odd inactive conformation using clues garnered from the other conformations they observed and from sophisticated computer simulations that allowed them to model changes from one configuration to another. The results of the simulations supported their speculation that the Src-like form might be an intermediate that facilitates the DFG flip.

Could this be a clue to imatinib resistance? Some forms of imatinib resistance are known to result from mutations that block the binding of imatinib to Abl. More commonly, however, mutations prevent Abl from adopting the conformation that imatinib binds to. The researchers noted that such imatinib-resistant mutations tend to destabilize the Src-like conformation of Abl more than the active or imatinib-bound conformations of Abl, suggesting that this conformation does indeed play a role in whether imatinib is effective in blocking the activity of Abl in CML cells.

## 

**Figure:**
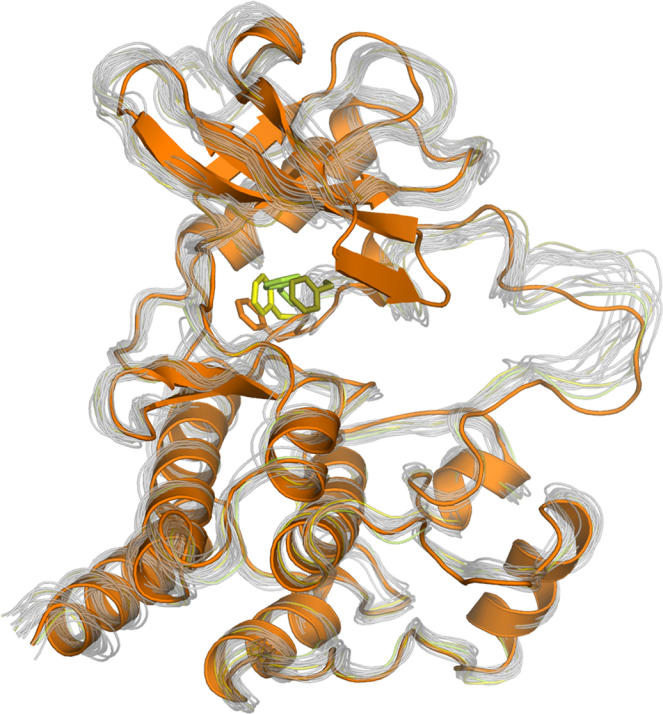
Simulations of the SCR-like inactive conformation of Abl

